# The effectiveness, efficiency, and acceptability of EMDR vs. EMDR 2.0 vs. the Flash technique in the treatment of patients with PTSD: study protocol for the ENHANCE randomized controlled trial

**DOI:** 10.3389/fpsyt.2023.1278052

**Published:** 2023-11-09

**Authors:** Valentijn V. P. Alting van Geusau, Ad de Jongh, Mae D. Nuijs, Thomas C. Brouwers, Mirjam Moerbeek, Suzy J. M. A. Matthijssen

**Affiliations:** ^1^Altrecht Academic Anxiety Center, Altrecht GGz, Utrecht, Netherlands; ^2^Academic Center for Dentistry Amsterdam, University of Amsterdam and VU University Amsterdam, Amsterdam, Netherlands; ^3^Psychotrauma Expertise Center (PSYTREC), Bilthoven, Netherlands; ^4^School of Health Sciences, University of Salford, Manchester, United Kingdom; ^5^Institute of Health and Society, University of Worcester, Worcester, United Kingdom; ^6^School of Psychology, Queen’s University, Belfast, Ireland; ^7^Department of Methodology and Statistics, Utrecht University, Utrecht, Netherlands

**Keywords:** EMDR, 2.0, Flash, PTSD, effectiveness, efficiency, acceptability, randomized controlled trial

## Abstract

**Background:**

Several widely studied therapies have proven to be effective in the treatment of post-traumatic stress disorder (PTSD). However, there is still room for improvement because not all patients benefit from trauma-focused treatments. Improvements in the treatment of PTSD can be achieved by investigating ways to enhance existing therapies, such as eye movement desensitization and reprocessing (EMDR) therapy, as well as exploring novel treatments. The purpose of the current study is to determine the differential effectiveness, efficiency, and acceptability of EMDR therapy, an adaptation of EMDR therapy, referred to as EMDR 2.0, and a novel intervention for PTSD, the so-called Flash technique. The second aim is to identify the moderators of effectiveness for these interventions. This study will be conducted among individuals diagnosed with PTSD using a randomized controlled trial design.

**Methods:**

A total of 130 patients diagnosed with (complex) PTSD will be randomly allocated to either six sessions of EMDR therapy, EMDR 2.0, or the Flash technique. The primary outcomes used to determine treatment effectiveness include the presence of a PTSD diagnosis and the severity of PTSD symptoms. The secondary outcomes of effectiveness include symptoms of depression, symptoms of dissociation, general psychiatric symptoms, and experiential avoidance. All patients will be assessed at baseline, at 4-week post-treatment, and at 12-week follow-up. Questionnaires indexing symptoms of PTSD, depression, general psychopathology, and experiential avoidance will also be assessed weekly during treatment and bi-weekly after treatment, until the 12-week follow-up. Efficiency will be assessed by investigating the time it takes both to lose the diagnostic status of PTSD, and to achieve reliable change in PTSD symptoms. Treatment acceptability will be assessed after the first treatment session and after treatment termination.

**Discussion:**

This study is the first to investigate EMDR 2.0 therapy and the Flash technique in a sample of participants officially diagnosed with PTSD using a randomized controlled trial design. This study is expected to improve the available treatment options for PTSD and provide therapists with alternative ways to choose a therapy beyond its effectiveness by considering moderators, efficiency, and acceptability.

**Trial registration:**

The trial was retrospectively registered in the ISRCTN registry at 10th November 2022 under registration number ISRCTN13100019.

## 1. Introduction

### 1.1. PTSD and treatment

Post-traumatic stress disorder (PTSD) is a trauma- and stressor-related disorder that can strongly interfere with daily functioning and is a significant public health burden (1, 2). Different treatments for PTSD, including eye movement desensitization and reprocessing (EMDR) therapy, prolonged exposure, and cognitive processing therapy have proven to be effective (3). EMDR therapy is currently recommended as one of the first-line treatments because of its strong evidence base (3–6). Although meta-analyses have found that EMDR therapy has large beneficial effects on PTSD compared to control conditions, and has large effect sizes in reducing PTSD symptoms (7, 8), there is still room for improvement. For instance, controlled outcome studies show that 5–64% of patients still meet the diagnostic criteria for PTSD after treated with EMDR therapy (4). Furthermore, 18% of patients dropped out before treatment with EMDR was completed and consequently did not benefit from the therapy (9). Therefore, it is imperative to investigate ways to improve current treatment methods for PTSD, and to find ways to make treatment more acceptable, aimed at reducing the attrition rate. One way to improve the therapeutic effectiveness is by optimizing existing evidence-based treatments, and another is by exploring new treatment methods.

### 1.2. Optimizing EMDR therapy: EMDR 2.0

Since the introduction of EMDR therapy by Shapiro (10), experimental research has focused on understanding its underlying working mechanisms [e.g., (11, 12)]. The results of these experimental research studies provide a theoretical framework that can be used to optimize the effectiveness of EMDR therapy. Although there are several different theories that might explain the effects of EMDR therapy, the working memory theory (13) currently has the strongest evidence base [e.g., (12, 14–16); for a more detailed overview of different theories explaining the working mechanisms of EMDR therapy, see Landin-Romero et al. (17)]. Recalling traumatic memory and tracking moving hands with the eyes are core elements of EMDR therapy. According to the working memory theory, these tasks both tax an individuals’ working memory. Because working memory capacity is limited (18), both tasks compete for the same resources. As a result of this dual task, the individual is not able to retain traumatic memory with the same emotionality and vividness, which in turn reduces the emotional impact and vividness of the memory. The memory is then reconsolidated in a modified state (13, 19, 20). Given that taxation of working memory is suggested to be responsible for the desensitization of aversive memories, it follows logically that a larger amount of working memory taxation is more effective in reprocessing such memories. In line with this idea, several studies have observed that the more the working memory is taxed (e.g., faster eye movements), the more effective EMDR is in degrading aversive memories (19, 21–24).

Furthermore, evidence suggests that adding modality-specific working memory taxation might improve EMDR effectiveness (25). However, it should be noted that the evidence of this modality-specific effect is still limited and contradictory (26). In addition, several studies have indicated that increased arousal can boost memory updating during reconsolidation (27–30). Furthermore, evidence suggests that adding an element of surprise could have a destabilizing effect on memories, rendering them vulnerable to modification via subsequent interference and influencing the reconsolidation process (31). The latter is also an assumed mechanism of action of another trauma focused treatment, visual schema displacement therapy (32, 33). In addition, some studies highlight the need for optimal activation of the traumatic memory while performing a dual task (34, 35). For example, Cuperus et al. (34) suggested that displaying a screenshot representing a negative memory while performing a dual task led to a greater decrease of the emotionality of the memory than only recalling the memory while performing a dual task. Conversely, another study found that continuous and deliberate memory recall in addition to working memory taxing dual-tasks did not increase reductions in emotionality and vividness of negative autobiographical memories, when compared to dual tasks only (36). These findings among participants without PTSD suggest that memory recall may not be a necessary element of EMDR therapy. Although the evidence is contradictory, Cuperus et al. (34) and van Veen et al. (35) have indicated that EMDR therapy may be improved by ensuring that traumatic memory is properly activated.

Based on the results of these studies, authors AJ and SM developed an alternative to the standard EMDR procedure (37, 38), which will henceforth be referred to as EMDR 2.0 (39). The EMDR 2.0 procedure incorporates knowledge derived from the results of the aforementioned experimental studies, with the purpose of enhancing the treatment effects of EMDR therapy. More specifically, EMDR 2.0 aims to increase working memory taxation and activation of traumatic memory, add arousal, add modality-specific working memory taxation, and an element of surprise (39). In a randomized controlled trial with a non-clinical sample, the effects of EMDR 2.0 have been tested by comparing a single session of standard EMDR therapy with a single session of EMDR 2.0 (39). No difference in efficacy was observed between EMDR and EMDR 2.0 in reducing the vividness and emotionality of aversive memories. However, EMDR 2.0 proved to be more efficient than EMDR therapy in reducing the emotionality of aversive memories. In a preliminary study with participants who experienced traffic accidents, a group protocol of EMDR 2.0 was investigated (40). The results showed that the EMDR 2.0 group protocol was effective in reducing the symptoms of PTSD and depression. The study by Matthijssen et al. (41) had a non-clinical sample, and the study by Yaşar et al. (40) did not have a sample of patients diagnosed with PTSD, nor did it have a control group. Therefore, it is not yet clear whether EMDR 2.0 would be effective and efficient in a sample of patients with a PTSD diagnosis.

### 1.3. The Flash technique

Similar to most other treatments for PTSD, EMDR therapy involves the recall of traumatic memories. For some patients this may pose a problem as they tend to avoid recalling their traumatic memories or dissociate when doing so. This is concerning because exposure to traumatic content is proposed to be essential during EMDR therapy to activate the traumatic memory network (38). Furthermore, difficulties in tolerating trauma-focused treatment have been suggested as reasons for dropout (9). For patients who find the recall of a traumatic memory too disturbing, the Flash technique has been developed since exposure to memory is minimized in this intervention (42).

During the application of the Flash technique, individuals are asked to select a disturbing memory or image as the target for treatment and rate its disturbance. They are discouraged from actively recalling details of the target as opposed to EMDR therapy and exposure-based treatments. The very brief exposure to the traumatic memory could in fact facilitate its processing (43). Subsequently, individuals are instructed to focus on a positive and engaging image or memory (positive engaging focus; PEF), which is intended to provide an immediate experience of pleasure. During this positive engaging focus, patients are discouraged from intentionally activating their targeted traumatic memory. Several times during the imagination of the PEF, the therapist says “Flash!.” Upon this prompt, patients are asked to blink rapidly several times while maintaining their attention to the PEF. After several prompts, patients are asked to briefly check in on their traumatic memory, notice any changes, and quickly rate the distress that it causes without fully tuning into the memory. Although the Flash technique was originally developed as a preparatory technique to improve tolerability for EMDR therapy, it has evolved into a stand-alone intervention [for a detailed description, see Manfield et al. (44)].

Several uncontrolled studies have been conducted that examined the effectiveness of the Flash technique. These studies suggest that the Flash technique is effective in reducing avoidance during recall of traumatic memories, disturbance or emotionality of traumatic memories, PTSD symptoms, symptoms of depression, and symptoms of dissociation (42, 44–47). However, as these studies did not have a control condition, random allocation to treatment conditions, or clinical interviews to assess PTSD diagnosis and treatment outcome, it is yet unclear whether the Flash technique would be effective in the treatment of PTSD. Until now, two controlled studies on the Flash technique have been conducted. Brouwers et al. (48) compared the Flash technique to an abbreviated version of the EMDR therapy protocol in non-clinical clinical participants, who were asked to recall an aversive autobiographical memory. Although no differences between EMDR and Flash were observed in reducing emotionality and vividness of aversive memories, participants rated the Flash technique as more pleasant than EMDR therapy. In another controlled study on traffic accident victims, the effect of the Flash technique was compared to a stress management module, which mainly consisted of psychoeducation (49). The study found that the Flash technique condition outperformed the stress management module condition in terms of reductions in anxiety, intrusions, and avoidance, and in improving general quality of life. Based on these results, it is conceivable that the Flash technique could be an effective intervention for PTSD, yet less burdensome than EMDR therapy.

### 1.4. Current study

The available literature shows promising results regarding both EMDR 2.0 therapy and the Flash technique. However, for EMDR 2.0 and the Flash technique, a randomized controlled trial with participants diagnosed with PTSD is still lacking. Therefore, the goal of the present study will be to investigate EMDR 2.0 and the Flash technique in a sample of participants diagnosed with PTSD, and to determine their relative effectiveness, when compared with each other and standard EMDR therapy (38) as an active control condition. We chose EMDR therapy as a comparator, because it is one of the first-choice interventions for PTSD according to the international treatment guidelines (50). This allows for direct comparison between the experimental conditions and the extensively studied EMDR therapy. The proposed study has three aims. Firstly, we will determine the differential effectiveness of EMDR therapy, EMDR 2.0, and the Flash technique. More specifically, we will investigate which of these treatment conditions is most effective in reducing (complex) PTSD symptoms and comorbid psychiatric symptoms, as well as the loss of the diagnostic status of PTSD. In addition, we will investigate whether EMDR 2.0 and the Flash technique are effective treatments, by inspecting changes in these outcomes over time. We will also determine the moderators of effectiveness. The moderators that will be investigated are PTSD symptom severity, symptoms of depression and dissociation, experiential avoidance, and the presence of a complex PTSD diagnosis. To this end, identifying potential moderators can help to indicate which treatment option is most suitable for different types of patient characteristics. Secondly, we will investigate the differential efficiency of the three treatments. Thirdly, we will investigate whether the treatments differ in terms of treatment acceptability.

There is not enough evidence to have *a priori* expectations with regards to the differential effectiveness of standard EMDR therapy compared to EMDR 2.0 or the Flash technique. Based on the results of earlier research on EMDR 2.0 and the Flash technique [e.g., (39, 48)], we expect that all interventions will be effective in terms of a significant reduction of (complex) PTSD symptoms and loss of diagnostic status. We also expect that all interventions will be effective in reducing the symptoms associated with PTSD. Considering the moderators, we expect that participants who show higher baseline symptoms of depression, will experience more difficulties in retrieving positive memories based on studies suggesting a negative memory bias in individuals with depression (51, 52). Therefore, we expect them to benefit less from the Flash technique than from EMDR therapy or EMDR 2.0. Furthermore, we expect participants who show relatively higher levels of experiential avoidance (defined as attempts to avoid thoughts, feelings, memories, physical sensations, and other internal experiences) and symptoms of dissociation at baseline, will benefit more from the Flash technique than from EMDR therapy. The reasoning underlying this hypothesis is that during the Flash technique only brief exposure to traumatic material is needed (42). Therefore, participants who are more avoidant, or tend to dissociate, will likely have less difficulty performing the Flash technique, than EMDR therapy. Based on the results of Matthijssen et al. (39), EMDR 2.0 is expected to be more efficient than standard EMDR therapy in treating traumatic memories and PTSD symptoms. Based on the preliminary findings of Brouwers et al. (48), we expect that the Flash technique will be rated as more acceptable by patients than standard EMDR therapy.

### 1.5. Design

The design of the current study is a parallel group, open, randomized controlled trial with three treatment arms with EMDR 2.0 and the Flash technique as experimental conditions and standard EMDR therapy as the active control condition, and an 1:1 allocation ratio. The three treatment conditions will be compared to each other on a number of outcome variables. We aim to complete the inclusion of participants within 3 years.

## 2. Methods and analysis

### 2.1. Participants

Participants will be recruited at the Altrecht Academic Anxiety Center in Utrecht, Netherlands. The Altrecht Academic Anxiety Center is an academic outpatient mental healthcare clinic, specialized in diagnosing and treating obsessive-compulsive disorders, anxiety disorders, and trauma-related disorders. The study participants will be patients diagnosed with PTSD who have applied for treatment. The inclusion criteria are as follows: a primary diagnosis of PTSD, a minimum age of 18 years old, an estimated IQ of > 80, and sufficient understanding of the Dutch language. The use of benzodiazepines, alcohol, and other drugs is contraindicated for treatment and will be tapered as much as possible before participation. Exclusion criteria are: acute suicidality [as assessed by a senior clinician, and using the suicidality module of the mini-international neuropsychiatric interview; MINI; (53)], following concurrent treatment for PTSD, and any changes in psychopharmacological medication during the study or 6 weeks prior to participation, with the exception of tapering benzodiazepines.

### 2.2. Interventions

#### 2.2.1. General treatment procedure

After treatment allocation and before the commencement of the treatment sessions, an individual treatment plan will be drawn up for each participant. For this treatment plan, patients will select several traumatic memories in collaboration with a therapist and will be requested to indicate the level of disturbance upon recalling a specific traumatic memory. This will be indexed using a subjective units of disturbance (SUD) scale with scores ranging from 0 to 10 (54). Traumatic memories are defined by the description of the A-criterion in the DSM-5 (55). The treatment proceeds in the order of memories with the highest SUD score. When a memory is desensitized to a SUD score of zero, treatment will proceed with the next memory on the treatment plan. If all A-criterion memories have been desensitized before the end of treatment, other disturbing memories will also be targeted. All treatment conditions consist of weekly, 60-min treatment sessions for a duration of 6 weeks. Completion of treatment is defined by completion of all six treatment sessions, or successful treatment (i.e., a SUD score of zero) for all traumatic memories as well as a loss of PTSD diagnostic status. The interventions will be discontinued if participants request so, or if the physical or mental state of the patient prohibits continuation of the intervention. Exacerbation of symptoms during treatment does not necessarily lead to exacerbation of symptoms after completion of treatment (56) and is therefore not a criterion for discontinuation.

#### 2.2.2. EMDR therapy

Therapists in the EMDR therapy condition use a Dutch version of the eight-phase protocol for EMDR therapy (38) (Supplementary Data Sheet 2). EMDR therapy uses a standardized procedure that requests patients to bring up a traumatic and disturbing memory, and concurrently engage in a dual-attention task. The tasks require patients to bring up their memory and, at the same time follow the therapist’s fingers with their eyes, thereby making horizontal movements. In practice, other tasks such as tones and hand taps may also be used. However, in the current study, we limit the task to making eye movements as much as possible, because eye movements have been proven to be most effective (57). For a full description of the treatment protocol, see Shapiro (38).

#### 2.2.3. EMDR 2.0 therapy

Eye movement desensitization and reprocessing (EMDR) 2.0 therapy is an adaptation of the standard procedure of EMDR therapy and is administered according to the EMDR 2.0 protocol (39) (Supplementary Data Sheet 1). One of the elements of EMDR 2.0 is the therapist’s effort to motivate patients for treatment by explaining the rationale of treatment and emphasizing the importance of placing the trauma memory as best and as fully as possible in their working memory. Thus, patients are active collaborators instead of passive receivers of treatment. As in standard EMDR therapy, participants are asked to describe their traumatic memory, select the most distressing image of that memory and rate its level of disturbance using a SUD score. A second key element is that activation of the trauma memory is enhanced by asking participants to focus on all sensory aspects of the traumatic memory, and/or by adding a stimulus that triggers the traumatic memory (34, 35). The third, and last, pillar of EMDR 2.0 is maximizing working memory taxation during the desensitization phase, by performing eye movements much faster than during standard EMDR therapy, supplemented with, if possible for the participant, at least one other task, such as tapping patterns or spelling words (19, 21–24). In addition, therapists aim to match the added working memory taxation to the modality of the traumatic memory which is most prominent (18, 25). For example, when a traumatic memory has a distressing auditory component, working memory is also taxed by asking participants to say and listen to certain words and sentences, in addition to making eye movements, thereby specifically taxing the auditory component of the working memory. Also, elements of surprise are added during desensitization by asking participants certain questions, such as: “What hair color do I have?” [i.e., disruption by surprise; (31)]. Finally, practitioners aim to increase participants’ arousal, for example, by suddenly clapping hands (27–30). Ideally, all of the above-mentioned components should be incorporated in the therapy session. However, therapists are trained to match the capacities of patients and may choose to leave out a component if they estimate it defeats its purpose. The rest of the procedure is identical to standard EMDR therapy (37, 38).

#### 2.2.4. The Flash technique

Participants in the Flash technique condition will undergo the Flash technique procedure (44) (Supplementary Data Sheet 3). During the Flash technique, participants are first requested to select a still image of a traumatic memory, which they will recall very briefly to determine its SUD score. Participants are encouraged to merely “touch on” their disturbing image, without recalling it vividly. Subsequently, they will select a positive memory or activity that provides an immediate experience of pleasure (the positive engaging focus or PEF). When engaging in the PEF, participants are asked to blink rapidly for five subsequent times. After blinking, therapists motivate the participants to keep activating the PEF. After five of such prompts of blinking, the SUD of the traumatic memory is determined, again without recalling it vividly. This procedure is repeated until the SUD reaches a score of 0. The procedure in this condition is identical to the procedure described by Brouwers et al. (48). The distraction component described in Manfield et al. (44) of alternating tapping on both thighs when engaging in the PEF is omitted.

#### 2.2.5. Treatment fidelity and therapist training

All treatment sessions will be performed by masters’ level psychologists who have completed at least a basic EMDR (“Level 1”) therapy course accredited by the EMDR Europe Association, and received additional consultation in order to demonstrate their competence. All therapists will attend an additional course provided by an accredited EMDR Europe trainer from the United Kingdom, in order to ensure the highest level of treatment fidelity in the application of the standard EMDR protocol (38). The EMDR Europe trainer will, together with another independent Dutch EMDR Europe trainer, also be involved in supervision and consultation of therapists throughout the study. Therapists who will perform the EMDR 2.0 and Flash technique sessions will attend two separate 1-day courses in these techniques, provided by two of the authors (AJ and SM). All therapists will be trained and providing treatment in all three treatment methods, limiting the influence of the therapist factor on treatment outcome. Treatment sessions of all conditions will be recorded on video. Recordings will be used for fidelity checks and treatment supervision. Protocol adherence will be tested using a therapy fidelity rating scale developed for this purpose. Also, all therapists will receive supervision by accredited consultants in the different methods. Consultants will also be involved in setting up the case conceptualization and are involved in looking over treatment fidelity using a secure social media platform (AJ and SM for EMDR 2.0 and the Flash technique and the Dutch EMDR Europe trainer for EMDR).

### 2.3. Procedure

#### 2.3.1. Recruitment and screening

The study will be conducted at the Altrecht Academic Anxiety Center. Individuals with PTSD symptoms will be approached by their therapists to participate in the current study. If written consent has been given, the research assistant may contact individuals directly as well. Those who are interested in participating will be invited for an appointment with one of the research assistants. During this appointment, the study will be explained and patients who wish to participate are asked to sign an informed consent-form. After consent, the Life Events Checklist for the DSM-5 (LEC-5) will be administered, along with a screening questionnaire assessing age, completed education, previously followed trauma-focused treatment, medication use, alcohol- and drug use, and suicidality. If patients report suicidal thoughts, suicidality risk will be assessed using the suicidality module of the MINI. If patients report a high risk of suicidality on the MINI., a senior therapist trained in the assessment of suicidality will make a final decision about the participation. By administering the Clinician-Administered PTSD Scale for DSM-5 (CAPS-5) and the screening questionnaire containing questions about the in- and exclusion criteria, it will be determined whether patients meet the criteria for participation.

#### 2.3.2. Randomization and allocation

After inclusion and the baseline assessment, participants will be randomly assigned to one of the three treatment conditions: EMDR therapy, EMDR 2.0, or the Flash technique, by means of permuted block randomization with block sizes of 3, 6 and 9 to be sure that group sizes are equal across conditions. Randomization will be performed by an independent researcher using an online tool. Stratified randomization will be performed with complex PTSD as stratification factor to ensure that patients with complex PTSD are equally distributed across conditions. There will be no blinding as participants and therapists will be able to tell which treatment arm they are allocated to. The CAPS-5 assessments, however, are conducted by assessors who are blind to the allocated treatment conditions.

#### 2.3.3. Assessments

**FIGURE 1:**
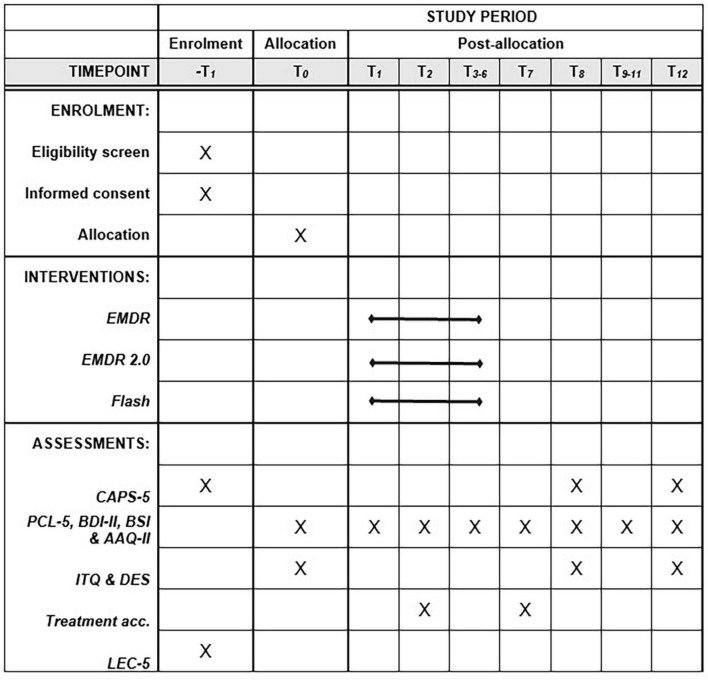
SPIRIT timeframe of the study. From T1 to T6, the measurements take place weekly. From T7 to T12, the measurements take place bi-weekly.

At baseline assessment (T0), the CAPS-5 will be administered by a trained therapist, along with the Life Events Checklist for DSM-5 (LEC-5). The International Trauma Questionnaire (ITQ), PTSD Checklist for DSM-5 (PCL-5), Dissociative Experiences Scale (DES), Beck Depression Inventory-II (BDI-II), Brief Symptom Inventory (BSI), and the Acceptance and Action Questionnaire-II (AAQ-II) will be administered digitally. The baseline assessment takes approximately 90 min. After the baseline assessment and the session where the treatment plan is drawn up, a 6-week treatment phase commences during which participants fill out questionnaires weekly prior to the treatment sessions (T1-6). Questionnaires include the PCL-5, BDI-II, BSI, and AAQ-II and take approximately 30 min in total to complete. After the first treatment session (T2), a questionnaire about treatment acceptability is administered. After the treatment phase, a 12-week phase commences where no actual treatment is delivered, but participants fill out the PCL-5, BDI-II, BSI, and AAQ-II bi-weekly (T7-12). The treatment acceptability questionnaire is administered again at T7. The CAPS-5, ITQ, and DES are administered 4 weeks after treatment is completed (T8), and 12 weeks after termination of treatment (T12). To minimize missing data, reminders will be sent out to the participants to fill out the questionnaires. For a complete overview of the timing of the interventions and assessments, see Figure 1.

### 2.4. Instruments

#### 2.4.1. Primary outcome measures

The primary study outcomes are severity of (complex) PTSD symptoms and PTSD diagnosis which are measured with several instruments.

##### 2.4.1.1. Clinician-Administered PTSD Scale for DSM-5 (CAPS-5)

The Dutch version of the CAPS-5 (58, 59) is a clinician rated structured interview to assess PTSD diagnosis and symptom severity over the past month. The severity of each symptom is scored on a 0–4 scale. A sum score is computed and can range from 0 to 80. The CAPS-5 is considered to be the golden standard for assessing PTSD and shows high internal consistency and modest interrater reliability (60).

##### 2.4.1.2. PTSD Checklist for DSM-5 (PCL-5)

The Dutch version of the PCL-5 (61, 62) will be used to measure PTSD symptom severity over the past week. The PCL-5 is a 20-item self-report measure with total scores ranging from 0 to 80, and is widely used in scientific papers, which improves the comparability of the results. The monthly version of the PCL-5 has good internal consistency, test-retest reliability, and convergent and discriminant validity (63). The weekly version of the PCL-5 is currently still undergoing psychometric evaluation (64).

##### 2.4.1.3. International Trauma Questionnaire (ITQ)

To assess complex PTSD (CPTSD) symptoms and diagnosis, the Dutch version of the ITQ (65, 66) will be used. The ITQ is an 18-item self-report measure with total scores ranging from 0 to 72. The ITQ has demonstrated high internal consistency and has shown to measure reliable and clinically significant treatment-related change of ICD-11 CPTSD symptoms (67).

#### 2.4.2. Secondary outcome measures

Secondary study parameters are comorbid symptoms such as depression, experiential avoidance, treatment acceptability, and moderators of treatment effectiveness, which are measured with several self-report measures.

##### 2.4.2.1. Beck Depression Inventory-II (BDI-II)

The Dutch version of the BDI-II (68, 69) is a self-report measure that assesses symptoms of depression with 21 items. Total scores range from 0 to 63. This score can be used to assess the presence and severity of symptoms of depression. The BDI-II has been shown to have good psychometric properties (69).

##### 2.4.2.2. Dissociative Experiences Scale (DES)

The Dutch version of the DES (70, 71) will be used to measure several symptoms of dissociation. It is a 28-item self-report measure with an average total score ranging from 0 to 100. A higher score is interpreted as a higher level of dissociation and a cut-off score of 25 is indicative for the presence of a dissociative disorder (72). The Dutch DES has good internal consistency and good criterion validity (71).

##### 2.4.2.3. Brief Symptom Inventory (BSI)

The Dutch version of the BSI (73, 74) is a 53 item self-report measure that assesses general psychiatric symptoms. Total scores range from 0 to 212 and can be used to indicate the severity of psychopathology on several dimensions. The BSI has shown good reliability and validity (75).

##### 2.4.2.4. Acceptance and Action Questionnaire-II (AAQ-II)

The Dutch version of the AAQ-II (76, 77) is a 7-item self-report measure that assesses experiential avoidance. Total scores range from 7 to 49. The Dutch AAQ-II has been demonstrated to have adequate reliability and variability (77).

##### 2.4.2.5. Treatment acceptability

Treatment acceptability is assessed with four questions based on Tarrier et al. (78) and Devilly (79) which measure several facets which could be grouped under treatment acceptability. Three questions are: “How did you experience treatment?” The answer options range from 1 (very unpleasant) to 7 (very pleasant), from 1 (very intrusive) to 7 (not intrusive at all), and from 1 (very tiring) to 7 (not tiring at all). The fourth question is: “How likely is it that you would recommend the treatment to someone with the same symptoms?,” with answer options that range from 1 (very unlikely) to 7 (very likely). As the questions combined have not been validated as a questionnaire measuring the full concept of treatment acceptability, the items will be scored and interpreted separately instead of computing a total score.

##### 2.4.2.6. Moderators

Moderators of treatment effectiveness to be investigated are: symptoms of dissociation, experiential avoidance, the presence of a complex PTSD diagnosis, PTSD symptom severity, and symptoms of depression. All moderators are assessed at baseline using the DES, AAQ-II, ITQ, CAPS-5, and BDI-II, respectively.

#### 2.4.3. Other measures

##### 2.4.3.1. Life Events Checklist for the DSM-5 (LEC-5)

The Dutch version of the LEC-5 (80, 81) is a 17-item questionnaire which measures the exposure to 16 different A-criterion events which are known to potentially result in PTSD, with an additional option for another stressful event. Answer options include: “Happened to me,” “witnessed it,” “learned about it,” “part of my job,” “not sure,” and “doesn’t apply.” It is used to assess the type of traumatic events participants have experienced. The psychometric properties of the LEC-5 have not yet been investigated. However, the LEC-5 differs little from the LEC for DSM-IV, which has adequate psychometric properties (82).

### 2.5. Sample size

To perform a power analysis for a multilevel model, it is required to estimate parameters based on earlier research (83), which was not possible for the interventions investigated in the current study. Power analyses were performed in G*Power 3.1.9.2, based on three pairwise comparisons between the conditions (EMDR therapy vs. EMDR 2.0, EMDR therapy vs. Flash, EMDR 2.0 vs. Flash) on the post-treatment measurement occasion. The power analysis is based on a two-sided *t*-test, in which two independent means will be compared with a Bonferroni-corrected alpha for the three pairwise comparisons = 0.0167 (0.05/3), power = 0.80 and Allocation ratio = 1. We aim to be able to detect a clinical meaningful difference between the conditions, what comes down to a medium to large effect size *d* = 0.65. To increase the power, we will include the baseline CAPS-5 score as a covariate in the analyses (84). By doing so, we expect to explain approximately a quarter of the variance at the post-treatment measurement. The residual variance reduces and gives us a sample size of *n* = 39 per condition, or *N* = 117 in total. To account for an expected 10% dropout, we will recruit a total 130 participants.

### 2.6. Data analyses

The statistical analyses will be performed on an intention-to-treat basis. Pre-treatment group differences are assessed using one-way ANOVAs for continuous data and χ2 tests for categorical data. A multilevel model will be performed with Treatment Condition (EMDR therapy vs. EMDR 2.0 vs. Flash technique) and Time (weekly assessments during the treatment and bi-weekly assessments during the follow-up phase) as predictors in the model. Measurements are nested within participants. Multilevel models are preferred over a repeated-measures ANOVA as participants with missing data can be included. In Level 1 of the model, the outcome measures vary within participants over time. In Level 2 of the model, the intercepts and slopes of the growth trajectories vary between participants. All analyses will be performed using the statistical programs R (Version 4.2.2) and IBM SPSS (Version 28).

#### 2.6.1. Aim 1: Treatment effectiveness

##### 2.6.1.1. Outcome measures CAPS-5, ITQ, and DES

To investigate the effectiveness of the three treatment conditions, the average scores will be estimated for the outcome variables at each time point for the three conditions. The baseline values will be included as covariate in the model. This will result in 3 (Condition: EMDR, EMDR 2.0, and Flash) × 2 (Time: T8 and T12) average score estimates. To investigate the differential effectiveness in reducing PTSD, complex PTSD and symptoms of dissociation of the treatments, these average score estimates will be compared across the three conditions. To answer the research question if the treatment arms are effective in reducing PTSD, complex PTSD, and symptoms of dissociation over time, the average scores of measurement occasions T0 vs. T8 and T0 vs. T12 will be compared using *t*-tests.

##### 2.6.1.2. Outcome measures PCL-5, BDI-II, BSI, and AAQ-II

For the outcome measures administered (bi)-weekly, a multilevel model will be performed. The baseline values will be included as covariate in the model. It will be investigated if the treatment conditions are effective in reducing self-reported PTSD symptoms (PCL-5), comorbid symptoms (BDI-II and BSI) and experiential avoidance (AAQ-II) over time. To investigate the differential effectiveness of the treatments, a Time × Condition interaction will be tested with the outcome measures as dependent variables. To investigate the effectiveness of the treatments, the effect of time will be tested with the outcome measures as dependent variables.

##### 2.6.1.3. Moderation analyses

To investigate whether treatment effect is moderated by baseline characteristics (baseline CAPS-5, PCL-5, DES, BDI-II, AAQ-II scores, and CPTSD diagnosis), Condition × Time × Moderator interaction effects will be tested with the primary outcome measures (CAPS-5, ITQ, and PCL-5) as dependent variables.

#### 2.6.2. Aim 2: Treatment efficiency

A survival analysis will be performed to investigate the efficiency of the interventions on symptom level. Estimates will be used to determine how much time is needed to reach a clinically significant change and a cut-off score on the PCL-5. Clinically significant change will be calculated by means of a Reliable Change Index (85). A cut-off score of 33 on the PCL-5 will be used, based on Bovin et al. (63) and Wortmann et al. (86). The estimates for the three treatment conditions will be compared using a log-rank test. A survival analysis is preferred since it accounts for censoring, which happens when not all participants reach a clinically significant change or the cut-off score during the course of the trial. Time will be defined by the amount of treatment sessions and ranges from one to six.

#### 2.6.3. Aim 3: Treatment acceptability

To investigate whether or not treatment acceptability differs between the treatment conditions, a one-way ANOVA with Treatment condition as the independent variable and scores on the individual treatment acceptability questions as dependent variables will be performed.

### 2.7. Data management and monitoring

To maintain confidentiality, data will be stored digitally on secured servers of the Altrecht Academic Anxiety Center. No analog data will be collected. Every participant will get an identification number, based on the order of inclusion. The data will be accessible by the principal investigator and research assistants involved in the study, affiliated with the Altrecht Academic Anxiety Center. Personal data will be handled according to the European General Data Protection Regulation. After consulting the guidelines posted by the European Medicines Agency, the installation of a Data Monitoring Committee was not deemed necessary. Pseudonymised data will be accessible to authors not affiliated with Altrecht. In accordance to the Dutch Medical Scientific Research Law, the trial will be suspended when there is sufficient ground that continuation of the study will jeopardize subjects’ health or safety. Serious adverse events (SAE’s) will be reported to the principal investigator within 24 h. SAE’s will also be reported to the Medical Research Ethics Committee (MREC) “NedMec” that approved the protocol.

## 3. Discussion

The current study may have several clinical implications. For instance, if our hypotheses that EMDR 2.0 and the Flash technique are effective treatments for PTSD are supported, it would offer therapists additional treatment options. Furthermore, if EMDR 2.0 is found to be more efficient than EMDR therapy, we would may be able to treat more patients within the same timeframe, which would reduce patient burden, as well as wait-list duration and treatment costs. This is necessary because PTSD is a major public health problem that incurs high healthcare costs (2). Another implication is that, if the Flash technique is found to be more acceptable than EMDR therapy, the application and dissemination of the Flash technique could reduce patient burden. Similarly, since treatment dissatisfaction appears to be related to treatment dropout, more acceptable treatments will likely improve treatment engagement and reduce attrition (78, 87, 88). Finally, the identification of moderators of treatment response would help match patients with the most suitable treatment options based on their characteristics.

The current study also has several scientific implications. One implication is that the current study is likely to provide a foundation for future research on EMDR 2.0 and the Flash technique, and their applicability in the clinical field, as well as future research on mechanisms of action. Furthermore, the present study could contribute to the collection of data on possible pre-treatment factors that moderate the effectiveness of PTSD treatment (89).

There are some limitations of the study design that need to be addressed. First, a limitation includes the risk of missing data, due to numerous measurement occasions after the treatment phase. However, by using a statistical method that handles missing data well, we mitigate the impact of missing data on the statistical power. Furthermore, a limitation of the present study is that the participants are not blinded to their treatment conditions. This makes the design susceptible to expectancy effects of the participants. However, we do not anticipate that these expectancy effects will be stronger for one condition than for another, and the randomization process should account for an even distribution if these effects would occur. Another limitation is the absence of an inactive control condition. To this end, on the one hand, our study is a suboptimal design for testing the hypotheses that the experimental conditions are effective treatments for PTSD. In contrast, the active control condition in this study has been frequently compared to wait-list control conditions (7, 8), showing that EMDR therapy was more effective in the treatment of PTSD than wait-list control conditions. Therefore, we can assume that when EMDR 2.0 and the Flash technique have comparable effect sizes in comparison with EMDR therapy, these interventions are likely to be more effective than a waitlist control condition in the treatment of PTSD. Finally, a limitation of the present study is that the design and sample size will not yield enough statistical power to detect small effect sizes when comparing the treatment conditions or when investigating moderators of effectiveness.

The study design also has several strengths. First, a wide array of outcomes of comorbid symptoms to PTSD (e.g., symptoms of depression and dissociation) will be assessed. Therefore, it is likely that the current study will add to the existing evidence of the effectiveness of EMDR therapy beyond PTSD symptoms (7, 8), and will allow us to compare EMDR 2.0 and the Flash technique with other trauma-focused treatments on these outcomes. Secondly, we will investigate whether the treatment results are maintained over a longer period, by monitoring the symptoms over the course of 12 weeks after treatment termination. Thirdly, therapists record their sessions on video and measure the time they spent on the intervention. Although this is originally incorporated in the procedure to assess treatment fidelity and treatment efficiency, this practice also serves to motivate therapists to optimally use the session time for the intervention and to not elaborate on peripheral matters, improving protocol adherence. Finally, we assess treatment efficiency not only using the intervention time needed to decrease subjective units of disturbance scores, but also by assessing the number of sessions needed to improve in symptomatology. Therefore, the current study may provide better insight into which treatment is most efficient in reducing PTSD symptoms and achieving clinically significant changes.

In conclusion, we believe that the proposed trial is of clinical and scientific relevance given that it is the first RCT to investigate EMDR 2.0 and the Flash technique in a sample of patients diagnosed with PTSD. In addition to focusing on effectiveness, the trial also investigates the treatment efficiency, acceptability, and moderators of effectiveness.

## Ethics statement

The studies involving humans were approved by the Medical Research Ethics Committee NedMec (NL79163.041.22). Important protocol modifications and study progress will be communicated to MREC NedMec according to their regulations. The studies were conducted in accordance with the local legislation and institutional requirements. The participants provided their written informed consent to participate in this study. A participant insurance was not deemed necessary by the MREC NedMec because of negligible risks associated with participation. Patients participating in the trial will not pay for the treatment they receive. Patients who still show symptoms after participation in the trial has been completed, will be offered additional treatment in our mental healthcare institution. We aim to publish the results of the current study in peer-reviewed journals, regardless of the outcome of the study. In addition, the results will be disseminated at national and international symposia and conferences. To be eligible for authorship, the authors will be required to have made substantive contributions to the design, analyses, conduct, interpretation, and/or reporting of the trial. Before inclusion, participants are informed of the study procedure using an information brochure. If they have had sufficient time to think (at least one week) and agree to participate, they will sign an informed consent form. Participants are informed of their right to withdraw from the study at any time. In accordance to the Dutch Medical Scientific Research Law, the trial will be suspended when there is sufficient ground that continuation of the study will jeopardize subjects’ health or safety. Serious adverse events (SAE’s) will be reported to the principal investigator within 24 h. SAE’s will also be reported to the Medical Research Ethics Committee (MREC) “NedMec” that approved the protocol.

## Author contributions

VA: Writing – original draft. AJ: Writing – review and editing. MN: Writing – review and editing. TB: Writing – review and editing. MM: Writing – review and editing. SM: Writing – review and editing.

## Trial status

Protocol version 1, date. Recruitment began at 17th October 2022 and will approximately be completed in October 2025.

## References

[1] Magruder KM McLaughlin KA Elmore Borbon DL. Trauma is a public health issue. *Eur J Psychotraumatol.* (2017) 8:1375338. 10.1080/20008198.2017.1375338 29435198 PMC5800738

[2] von der Warth R Dams J Grochtdreis T König HH. Economic evaluations and cost analyses in posttraumatic stress disorder: a systematic review. *Eur J Psychotraumatol.* (2020) 11:1753940. 10.1080/20008198.2020.1753940 33488993 PMC7803086

[3] Lewis C Roberts NP Andrew M Starling E Bisson JI. Psychological therapies for post-traumatic stress disorder in adults: Systematic review and meta-analysis. *Eur J Psychotraumatol.* (2020) 11:1729633. 10.1080/20008198.2020.1729633 32284821 PMC7144187

[4] De Jongh A Amann BL Hofmann A Farrell D Lee CW. The status of EMDR therapy in the treatment of posttraumatic stress disorder 30 years after its introduction. *J EMDR Pract Res.* (2019) 13:261–9. 10.1891/1933-3196.13.4.261 11261958

[5] Matthijssen SJMA Lee CW de Roos C Barron IG Jarero I Shapiro E The current status of EMDR therapy, specific target areas, and goals for the future. *J EMDR Pract Res.* (2020) 14:241–84. 10.1891/EMDR-D-20-00039 11261958

[6] De Jongh A ten Broeke E Farrell D Maxfield L. Empirically supported psychological treatments: EMDR therapy. In: Gayle Beck J Sloan DM editors. *The Oxford Handbook of Traumatic Stress Disorders, Second Edition.* Oxford: Oxford Academic (2020).

[7] Cuijpers P Veen SCV Sijbrandij M Yoder W Cristea IA. Eye movement desensitization and reprocessing for mental health problems: a systematic review and meta-analysis. *Cogn Behav Ther.* (2020) 49:165–80. 10.1080/16506073.2019.1703801 32043428

[8] Yunitri N Chu H Kang XL Wiratama BS Lee T-Y Chang L-F Comparative effectiveness of psychotherapies in adults with posttraumatic stress disorder: a network meta-analysis of randomized controlled trials. *Psychol Med.* (2023) 53:6376–88. 10.1017/S0033291722003737 36628572

[9] Lewis C Roberts NP Gibson S Bisson JI. Dropout from psychological therapies for post-traumatic stress disorder (PTSD) in adults: systematic review and meta-analysis. *Eur J Psychotraumatol.* (2020) 11:1709709. 10.1080/20008198.2019.1709709 32284816 PMC7144189

[10] Shapiro F. Efficacy of the eye movement desensitization procedure in the treatment of traumatic memories. *J Traumatic Stress.* (1989) 2:199–223. 10.1002/jts.2490020207

[11] Schubert SJ Lee CW Drummond PD. The efficacy and psychophysiological correlates of dual-attention tasks in eye movement desensitization and reprocessing (EMDR). *J Anxiety Disord.* (2011) 25:1–11. 10.1016/j.janxdis.2010.06.024 20709492

[12] van den Hout MA Engelhard IM Beetsma D Slofstra C Hornsveld H Houtveen J EMDR and mindfulness. Eye movements and attentional breathing tax working memory and reduce vividness and emotionality of aversive ideation. *J Behav Ther Exp Psychiatry.* (2011) 42:423–31. 10.1016/j.jbtep.2011.03.004 21570931

[13] Gunter RW Bodner GE. How eye movements affect unpleasant memories: support for a working-memory account. *Behav Res Ther.* (2008) 46:913–31. 10.1016/j.brat.2008.04.006 18565493

[14] De Jongh A Ernst R Marques L Hornsveld H. The impact of eye movements and tones on disturbing memories involving PTSD and other mental disorders. *J Behav Ther Exp Psychiatry.* (2013) 44:477–83. 10.1016/j.jbtep.2013.07.002 23892070

[15] Engelhard I van Uijen S van den Hout M. The impact of taxing working memory on negative and positive memories. *Eur J Psychotraumatol.* (2010) 1:5623. 10.3402/ejpt.v1i0.5623 22893797 PMC3402003

[16] van den Hout MA Engelhard IM Smeets MA Hornsveld H Hoogeveen E de Heer E Counting during recall: taxing of working memory and reduced vividness and emotionality of negative memories. *Appl Cogn Psychol.* (2010) 24:303–11. 10.1002/acp.1677

[17] Landin-Romero R Moreno-Alcazar A Pagani M Amann BL. How does eye movement desensitization and reprocessing therapy work? A systematic review on suggested mechanisms of action. *Front Psychol.* (2018) 9:1395. 10.3389/fpsyg.2018.01395 30166975 PMC6106867

[18] Baddeley AD Hitch G. Working memory. *Psychol Learn Motiv.* (1974) 8:47–89. 10.1016/S0079-7421(08)60452-1

[19] Maxfield L Melnyk WT Hayman GC. A working memory explanation for the effects of eye movements in EMDR. *J EMDR Prac Res.* (2008) 2:247–61. 10.1891/1933-3196.2.4.247 11261958

[20] van den Hout MA Engelhard IM. How does EMDR work? *J Exp Psychopathol.* (2012) 3:724–38. 10.5127/jep.028212

[21] De Voogd LD Phelps EA. A cognitively demanding working-memory intervention enhances extinction. *Sci Rep.* (2020) 10:1–11. 10.1038/s41598-020-63811-0 32341373 PMC7184585

[22] Littel M van Schie K. No evidence for the inverted U-Curve: more demanding dual tasks cause stronger aversive memory degradation. *J Behav Ther Exp Psychiatry.* (2019) 65:101484. 10.1016/j.jbtep.2019.101484 31125845

[23] van Schie K van Veen SC Engelhard IM Klugkist I van den Hout MA. Blurring emotional memories using eye movements: individual differences and speed of eye movements. *Eur J Psychotraumatol.* (2016) 7:29476. 10.3402/ejpt.v7.29476 27387843 PMC4933794

[24] van Veen SC van Schie K Wijngaards-de Meij LD Littel M Engelhard IM van den Hout MA. Speed matters: Relationship between speed of eye movements and modification of aversive autobiographical memories. *Front Psychiatry.* (2015) 6:45. 10.3389/fpsyt.2015.00045 25904871 PMC4387929

[25] Matthijssen SJMA van Schie K van den Hout MA. The Effect of modality specific interference on working memory in recalling aversive auditory and visual memories. *Cogn Emot.* (2018) 33:1169–80. 10.1080/02699931.2018.1547271 30465479

[26] Mertens G Lund M Engelhard IM. The effectiveness of dual-task interventions for modulating emotional memories in the laboratory: A meta-analysis. *Acta Psychol.* (2021) 220:103424. 10.1016/j.actpsy.2021.103424 34619553

[27] Anderson AK Yamaguchi Y Grabski W Lacka D. Emotional memories are not all created equal: evidence for selective memory enhancement. *Learn Mem.* (2006) 13:711–8. 10.1101/lm.388906 17101871 PMC1783624

[28] Littel M Remijn M Tinga AM Engelhard IM van den Hout MA. Stress enhances the memory-degrading effects of eye movements on emotionally neutral memories. *Clin Psychol Sci.* (2017) 5:316–24. 10.1177/2167702616687292

[29] Stein M Rohde KB Henke K. Focus on emotion as a catalyst of memory updating during reconsolidation. *Behav Brain Sci.* (2015) 38:e27. 10.1017/S0140525X14000314 26050691

[30] van den Hout MA Eidhof MB Verboom J Littel M Engelhard IM. Blurring of emotional and non-emotional memories by taxing working memory during recall. *Cogn Emot.* (2014) 28:717–27. 10.1080/02699931.2013.848785 24199660

[31] Sinclair AH Barense MD. Surprise and destabilize: prediction error influences episodic memory reconsolidation. *Learn Mem.* (2018) 25:369–81. 10.1101/lm.046912.117 30012882 PMC6049395

[32] Matthijssen SJMA van Beerschoten LM De Jongh A Klugkist IG van den Hout MA. Effects of “visual schema displacement therapy” (VSDT), an abbreviated EMDR protocol and a control condition on emotionality and vividness of aversive memories: two critical analogue studies. *J Behav Ther Exp Psychiatry.* (2019) 63:48–56. 10.1016/j.jbtep.2018.11.006 30514434

[33] Matthijssen SJMA Brouwers TC van den Hout MA Klugkist IG De Jongh A. A randomized controlled dismantling study of Visual Schema Displacement Therapy (VSDT) vs an abbreviated EMDR protocol vs a non-active control condition in individuals with disturbing memories. *Eur J Psychotraumatol.* (2021) 12:1883924. 10.1080/20008198.2021.1883924 33889309 PMC8043526

[34] Cuperus AA Laken M van Schie K Engelhard IM van den Hout MA. Dual-tasking during recall of negative memories or during visual perception of images: effects on vividness and emotionality. *J Behav Ther Exp Psychiatry.* (2019) 62:112–6. 10.1016/j.jbtep.2018.10.003 30316043

[35] van Veen SC Engelhard IM van den Hout MA. The effects of eye movements on emotional memories: Using an objective measure of cognitive load. *Eur J Psychotraumatol.* (2016) 7:30122. 10.3402/ejpt.v7.30122 27387845 PMC4933790

[36] van Schie K van Veen SC. Omitting continuous memory recall from dual-task interventions does not reduce intervention effectiveness. *Behav Res Ther.* (2023) 164:104291. 10.1016/j.brat.2023.104291 36933473

[37] De Jongh A Ten Broeke E. *Handboek EMDR: Een geprotocolleerde behandelmethode voor de gevolgen van psychotrauma* [Handbook EMDR: A protocol-based treatment method for the consequences of psychotrauma]. Amsterdam: Pearson Assessment and Information (2019).

[38] Shapiro F. *Eye Movement Desensitization and Reprocessing: Basic Principles, Protocols, and Procedures.* 3rd ed. New York: Guilford Press (2018).

[39] Matthijssen SJMA Brouwers TC van Roozendaal C Vuister T De Jongh A. The effect of EMDR versus EMDR 2.0 on emotionality and vividness of aversive memories in a non-clinical sample. *Eur J Psychotraumatol.* (2021) 12:1956793. 10.1080/20008198.2021.1956793 34567439 PMC8462855

[40] Yaşar AB Kavakçı Ö Çiftçi ZZ Tunca GA Uygun E Gündoğmuş İ The effectiveness of online EMDR 2.0 group protocol on posttraumatic stress disorder symptoms, depression, anxiety, and stress in individuals who have experienced a traffic accident: a preliminary study. *J EMDR Pract Res.* (2023) 17.

[41] Matthijssen SJMA Verhoeven LC Van den Hout MA Heitland I. Auditory and visual memories in PTSD patients targeted with eye movements and counting: the effect of modality-specific loading of working memory. *Front Psychol.* (2017) 8:1937. 10.3389/fpsyg.2017.01937 29163311 PMC5675874

[42] Manfield PE Lovett J Engel L Manfield D. Use of the flash technique in EMDR therapy: Four case examples. *J EMDR Pract Res.* (2017) 11:195–205. 10.1891/1933-3196.11.4.195 11261958

[43] Siegel P Warren R Wang Z Yang J Cohen D Anderson JF Less is more: neural activity during very brief and clearly visible exposure to phobic stimuli. *Hum Brain Mapp.* (2017) 38:2466–81. 10.1002/hbm.23533 28165171 PMC5385151

[44] Manfield PE Engel L Greenwald R Bullard DG. The flash technique in a low-intensity group trauma intervention for healthcare providers impacted by COVID-19 patients. *J EMDR Pract Res.* (2021) 15:127–39. 10.1891/EMDR-D-20-00053 11261958

[45] Gustavson K Wong SL Le D. Research on low-intensity flash technique trauma intervention by prelicensed student clinicians. *J EMDR Pract Res.* (2023) 17:54–69. 10.1891/EMDR-2022-0059 11261958

[46] Wong SL. Flash technique group protocol for highly dissociative clients in a homeless shelter: A clinical report. *J EMDR Pract Res.* (2019) 13:20–31. 10.1891/1933-3196.13.1.20 11261958

[47] Yaşar AB Gündoğmuş İ Gündüz A Konuk E. The effects of single session EMDR flash technique group application on traumatic symptoms. *Israel J Psychiatry.* (2021) 58:41–6.

[48] Brouwers TC De Jongh A Matthijssen SJMA. The effects of the Flash technique compared to those of an abbreviated eye movement desensitization and reprocessing therapy protocol on the emotionality and vividness of aversive memories. *Front Psychol.* (2021) 5845:741163. 10.3389/fpsyg.2021.741163 35002841 PMC8732365

[49] Yaşar AB Konuk E Kavakçı Ö Uygun E Gündoğmuş İ Taygar AS A randomized-controlled trial of EMDR flash technique on traumatic symptoms, depression, anxiety, stress, and life of quality with individuals who have experienced a traffic accident. *Front Psychol.* (2022) 13:1127. 10.3389/fpsyg.2022.845481 35401305 PMC8987710

[50] International Society of Traumatic Stress Studies. *New ISTSS Prevention and Treatment Guidelines.* (2018). Available online at: http://www.istss.org/treating-trauma/new-istssguidelines.aspx (accessed September 30, 2022).

[51] LeMoult J Gotlib IH. Depression: a cognitive perspective. *Clin Psychol Rev.* (2019) 69:51–66. 10.1016/j.cpr.2018.06.008 29961601 PMC11884012

[52] Matt GE Vázquez C Campbell WK. Mood-congruent recall of affectively toned stimuli: A meta-analytic review. *Clin Psychol Rev.* (1992) 12:227–55. 10.1016/0272-7358(92)90116-P

[53] Sheehan DV Lecrubier Y Sheehan KH Amorim P Janavs J Weiller E The Mini-International Neuropsychiatric Interview (MINI): the development and validation of a structured diagnostic psychiatric interview for DSM-IV and ICD-10. *J Clin Psychiatry.* (1998) 59:22–33. 9881538

[54] Wolpe J. *The Practice of Behavior Therapy.* 4th ed. New York, NY: Pergamon Press (1990).

[55] American Psychiatric Association. *Diagnostic and Statistical Manual of Mental Disorders.* 5th ed. Washington, DC: American Psychiatric Association (2013).

[56] Burger SR Hardy A van der Linden T van Zelst C de Bont PA van der Vleugel B The bumpy road of trauma-focused treatment: posttraumatic stress disorder symptom exacerbation in people with psychosis. *J Traumatic Stress.* (2023) 36:299–309. 10.1002/jts.22907 36719408

[57] Houben STL Otgaar H Roelofs J Merckelbach H Muris P. The effects of eye movements and alternative dual tasks on the vividness and emotionality of negative autobiographical memories: A meta-analysis of laboratory studies. *J Exp Psychopathol.* (2020) 11:2043808720907744. 10.1177/2043808720907744

[58] Boeschoten MA Bakker A Jongedijk RA van Minnen A Elzinga BM Rademaker AR *The Clinician-Administered PTSD Scale for DSM-5 (CAPS-5).* Diemen: Nederlandse Vertaling (2014).

[59] Weathers FW Blake DD Schnurr PP Kaloupek DG Marx BP Keane TM. *The Clinician-Administered PTSD Scale for DSM-5 (CAPS-5; past-month version).* (2013). Available online at: www.ptsd.va.gov (accessed September 30, 2022).

[60] Boeschoten MA van der Aa N Bakker A Ter Heide FJJ Hoofwijk MC Jongedijk RA Development and evaluation of the Dutch clinician-administered PTSD scale for DSM-5 (CAPS-5). *Eur J Psychotraumatol.* (2018) 9:1546085. 10.1080/20008198.2018.1546085 PMC6263102 30510643

[61] Boeschoten MA Bakker A Jongedijk RA Olff M. *The PTSD Checklist for DSM-5 (PCL-5).* Diemen: Nederlandse Vertaling (2014).

[62] Weathers FW Litz BT Keane TM Palmieri PA Marx BP Schnurr PP. *The PTSD Checklist for DSM-5 (PCL-5).* (2013). Available online at: www.ptsd.va.gov (accessed September 30, 2022).

[63] Bovin MJ Marx BP Weathers FW Gallagher MW Rodriguez P Schnurr PP Psychometric properties of the PTSD checklist for diagnostic and statistical manual of mental disorders–fifth edition (PCL-5) in veterans. *Psychol Assess.* (2016) 28:1379. 10.1037/pas0000254 26653052

[64] van der Does FHS Boeschoten M Baas M Covers M Giltay EJ ter Heide JJ *Methods of Assessing Post-Traumatic Stress Disorder Symptoms using the Dutch PCL-5 – Comparing Dichotomous, Categorical, and Network Approaches* [Manuscript in preparation]. Leiden: Leiden University Medical Center, Leiden University (2023).

[65] Cloitre M Shevlin M Brewin CR Bisson J I Roberts NP Maercker A The international trauma questionnaire: development of a self-report measure of ICD-11 PTSD and complex PTSD. *Acta Psychiatr Scand.* (2018) 138:536–46. 10.1111/acps.12956 30178492

[66] Eidhof M Ter Heide F Boeschoten M Olff M. *Internationale Trauma Vragenlijst: Zelfrapportage Vragenlijst Voor ICD-11 PTSS en CPTSS. Nederlandstalige Versie.* (2018). Available online at: http://www.psychotraumadiagnostics.centrum45.nl/ (accessed September 30, 2022).

[67] Cloitre M Hyland P Prins A Shevlin M. The international trauma questionnaire (ITQ) measures reliable and clinically significant treatment-related change in PTSD and complex PTSD. *European J Psychotraumatol.* (2021) 12:1930961. 10.1080/20008198.2021.1930961 34211640 PMC8221157

[68] Beck AT Steer RA Brown GK. *Manual for the Beck Depression Inventory-II.* San Antonio, TX: Psychological Corporation (1996).

[69] van der Does AJW. *BDI-II-NL. Handleiding. De Nederlandse versie van de Beck Depression Inventory-2nd edition.* Lisse: Harcourt Test Publishers (2002).

[70] Bernstein EM Putnam FW. Development, reliability, and validity of a dissociation scale. *J Nervous Ment Dis.* (1986) 174:727–35. 10.1097/00005053-198612000-00004 3783140

[71] Ensink BJ Van Otterloo D. A validation study of the DES in the Netherlands. *Dissoc Prog Dissoc Disord.* (1989) 2:221–3.

[72] Putnam FW Carlson EB Ross CA Anderson G Clark P Torem M Patterns of dissociation in clinical and nonclinical samples. *J Nervous Ment Dis.* (1996) 184:673–9. 10.1097/00005053-199611000-00004 8955680

[73] de Beurs E. *Brief Symptom Inventory. Handleiding.* Leiden: PITS (2006).

[74] Derogatis LR Melisaratos N. The brief symptom inventory: an introductory report. *Psychol Med.* (1983) 13:595–605. 10.1017/S0033291700048017 6622612

[75] de Beurs E Zitman FG. The Brief Symptom Inventory (BSI): reliability and validity of a practical alternative to SCL-90. *Maandblad Geestelijke Volksgezondheid.* (2006) 61:120–41.

[76] Bond FW Hayes SC Baer RA Carpenter KM Guenole N Orcutt HK Preliminary psychometric properties of the acceptance and action questionnaire–II: a revised measure of psychological inflexibility and experiential avoidance. *Behav Ther.* (2011) 42:676–88. 10.1016/j.beth.2011.03.007 22035996

[77] Jacobs N Kleen M De Groot F. Het meten van experiëntiële vermijding: de nederlandstalige versie van de acceptance and action ouestionnaire-II (AAQ-II). *Gedragstherapie.* (2008) 41:349–61.

[78] Tarrier N Liversidge T Gregg L. The acceptability and preference for the psychological treatment of PTSD. *Behav Res Ther.* (2006) 44:1643–56. 10.1016/j.brat.2005.11.012 16460671

[79] Devilly GJ. An approach to psychotherapy toleration: the Distress/Endorsement Validation Scale (DEVS) for clinical outcome studies. *J. Behav. Ther. Exp. Psychiatry* (2004) 35:319–36. 10.1016/j.jbtep.2004.08.001 15530846

[80] Boeschoten MA Bakker A Jongedijk RA Olff M. *The Life Events Checklist for DSM-5 (LEC-5).* Diemen: Nederlandse Vertaling (2014).

[81] Weathers FW Blake DD Schnurr PP Kaloupek DG Marx BP Keane TM. *The Life Events Checklist for DSM-5 (LEC-5).* (2013). Available online at: www.ptsd.va.gov (accessed September 30, 2022).

[82] Gray MJ Litz BT Hsu JL Lombardo TW. Psychometric properties of the life events checklist. *Assessment.* (2004) 11:330–41. 10.1177/1073191104269954 15486169

[83] Moerbeek M Teerenstra S. *Power Analysis of Trials With Multilevel Data.* Boca Raton: CRC Press (2015).

[84] Kahan BC Jairath V Doré CJ Morris TP. The risks and rewards of covariate adjustment in randomized trials: an assessment of 12 outcomes from 8 studies. *Trials.* (2014) 15:1–7. 10.1186/1745-6215-15-139 24755011 PMC4022337

[85] Jacobson NS Truax P. Clinical significance: a statistical approach to defining meaningful change in psychotherapy research. *J Consult Clin Psychol.* (1991) 59:12–9. 10.1037/10109-042 2002127

[86] Wortmann JH Jordan AH Weathers FW Resick PA Dondanville KA Hall-Clark B Psychometric analysis of the PTSD Checklist-5 (PCL-5) among treatment-seeking military service members. *Psychol Assess.* (2016) 28:1392. 10.1037/pas0000260 26751087

[87] Bados A Balaguer G Saldaña C. The efficacy of cognitive–behavioral therapy and the problem of drop-out. *J Clin Psychol.* (2007) 63:585–92. 10.1002/jclp.20368 17457848

[88] Swift JK Callahan JL. The impact of client treatment preferences on outcome: a meta-analysis. *J Clin Psychol.* (2009) 65:368–81. 10.1002/jclp.20553 19226606

[89] Cusack K Jonas DE Forneris CA Wines C Sonis J Middleton JC Psychological treatments for adults with posttraumatic stress disorder: A systematic review and meta-analysis. *Clin Psychol Rev.* (2016) 43:128–41. 10.1016/j.cpr.2015.10.003 26574151

