# Supplementary material for: The effectiveness, efficiency, and acceptability of EMDR vs. EMDR 2.0 vs. the Flash technique in the treatment of patients with PTSD: study protocol for the ENHANCE randomized controlled trial
Source: Front Psychiatry. 2023 Nov 9;14:1278052. doi: 10.3389/fpsyt.2023.1278052 (PMC10665892; doi:10.3389/fpsyt.2023.1278052)
Supplement: Supplementary file 1 [file Data_Sheet_1.pdf]

EMDR 2.0 PROTOCOL

1. Introduction

EMDR is applied when:

*The issues are known.

*The target memory has been determined.

*Any avoidance behaviors, situations and / or future fears have been mapped[1] to determine if *Flashforwards, Mental Video Checks* and *Future Templates must first be applied*).

"I'm going to ask some questions about your memory of the event we're going to work on. At some point, I'll ask you to focus on certain aspects of the memory. In particular, I will ask you about the image in your head that still evokes the most disturbance when you look at it. Only if you really zoom in on the worst aspects of that image as well as possible can it be changed, so don't shy away from anything. The most important thing is that you <u>really</u> bring the most unpleasant image of the memory into your working memory. Not just a little, but completely. Then I'm going to ask you to do two or more things at once. It will be to think about your bad memory tapping a rhythm on your legs and at the same time following my fingers moving back and forth quickly in front of your face, and probably some other things as well. Doing two or more of these things at the same time doesn't work very well, so you won't be able to hold onto that bad memory with the same bad feeling. In this way, that memory gradually becomes more and more vague, and less and less emotional."

[1] Check before starting the protocol if there is a strong self-reproach concerning the memory to be processed by asking, "Is self-reproach or guilt still playing a role in relation to this event at this moment?" If yes, "Is that self-reproach or guilt prominent in your perspective?" If so: "What image of the memory currently triggers the self-reproach or guilt the most at this moment?" Then, you can proceed directly to NC selection (skip to step 2.2: choice between self-esteem or guilt domains).

Introduce the super-fast eye movements and teach the complicated taps alternating on both legs. Pay attention to the position of the seats, the background, the speed, and the distance. If another distracting task is used, such as the light bar, the headphones, or buzzers, demonstrate their use. Everything must be ready in advance and demonstrated.

"In a moment I'll ask you to do your best to think as much as possible about the picture that represents the worst part of the memory, with all the details that come with it. Then I'm going to distract you as much as I can, so be sure to focus your attention on the memory as intensely as you can while performing the other tasks. As I said, it is very difficult to stay focused on the memory. That doesn't matter at all; it is precisely the intention. In the meantime, anything might come up. It could be thoughts, feelings, images, emotions, or physical sensations. Anything that comes up is fine. Just notice what comes up, without trying to control it and without asking yourself if you are doing it correctly. I will occasionally ask you to check the memory again to see how much disturbance you still feel. It's impossible to get it wrong. Just focus your attention on the memory image and what comes up. I will guide you through the process. Let's get started."

2. 'Focusing' (Assessment)

“Now close your eyes and don't open them until I ask you to... okay?"

(Skip this step for very anxious or dissociative people, or if they refuse to close their eyes).

2.1 Traumatic Memory

a. Visual representation of the negative experience

"Now tell me the bad memory broadly, from the point where you feel it starts, to the point where you feel it really ends. Describe the entire memory but not in great detail. It's about what your perceptions are and not so much about what exactly happened."

Let them recount the entire memory and assist from time to time, if necessary, by asking in a curious tone: ...*"And how does the memory continue...?"* until the actual end of the memory is reached.

If they do not want to share the details or cannot talk about the memory, say:

"You don't have to tell me anything about it, just concentrate deeply on the worst details. Can we agree on this?"

(Blind2Therapist mode).

Check:

Did any other things happen that you feel are part of it, either before or after?

b. Target Selection

Choose between the movie or the photo album metaphor (both are good choices).

Movie Metaphor[2]

"You just told me how this memory is stored in your head. Now notice: What picture represents the worst part of the memory at this moment, if you look at it from the here and now? Look at it as if it were a movie and then freeze it – at that second - so that it becomes a picture. We are mainly looking for a picture in which you can see yourself. It's not about what evoked the most distress at that time, but about the picture that is the most disturbing to you right now, including anything that didn't show up in the movie until later."[3] "What does the picture look like right now, that is the most disturbing for you at this moment?"

Or:

Photo album metaphor[2]

Suppose there is a photo album in your head, in which there are photos that show how you *currently remember this event. These are mainly photos in which you appear. It may be that there are some photos in that photo album that show things that did not actually happen but have only appeared in the photo album later.* [4]
So, which picture now, right now, is the most disturbing when you look at it?... "What do you see in that picture?

[2] Note: The client might not always see themselves in the image (for example, if they were 'only' a spectator at the time). If they do not automatically describe themselves in the image/photo even though in fact they did play a role by either doing, or not doing something, ask: *"Where do you see yourself in the picture/in the photo"?* Thus, it becomes a description of a still image which includes them.

[3] If it is an event that could have had a much worse outcome, ask explicitly: *"Is there perhaps a picture in the movie that shows what could have happened, in the worst case?"*

[4] If it concerns an event that could have turned out much worse, ask explicitly, "Is there perhaps a photo in the photo album showing what could have happened in the worst case scenario?"

If there are multiple disturbing images, help the patient choose:

"*Bring both pictures to mind, hang them side by side in your head, as it were, like on a clothesline. Which of those two pictures disturbs you the most right now, at this moment?*

Check:

*"Just to be sure; is this the image in your head that is the most disturbing when you look at it **now, right now**, rather than what you might have thought was the worst at the time?"*

Ask yourself, while paying attention to their story and the chosen target, in which category of clinical themes the negative cognition can most likely be found:

- Control (e.g., "I am powerless/helpless")
- Safety (concerning the situation, e.g. "I am emotionally still in danger" I can't trust.)
- Self-esteem (e.g., "I'm worthless, a bad person, a weakling, a coward")
- Responsibility/ guilt (e.g. "I am guilty. It's my fault.")

2.2 Negative cognition (NC)

"When you bring that image back to mind, does it feel disturbing to look at it right now, like you can't stand to look at it?

To speed up the process, we assume here that a standard negative cognition 'I can't stand it.' or 'I can't handle it.' applies, meaning that they can't handle looking at the picture. This negative cognition fits the clinical theme of control. If they concur, continue with 2.5, determining the emotion, as the PC has now been determined.

However, if you think there is a more valid negative cognition, select it instead. Listen carefully to identify clinical themes and continue questioning until you identify another possible category such as “Safety " (see A), ‘Self-esteem' or 'Guilt' (see B):

"What we need to figure out now is why this image in your mind still evokes so much tension when you look at it, at this moment, regardless of how distressing it was for you back then. What is it that makes this image still evoke so much tension now?"

A. Safety

If you suspect that it concerns a current lack of safety (i.e., the patient's body reacts to this memory as if the danger is still there), then say:

"Is it true that you feel as if you are in danger again when you look at the picture now?"

If this is correct, continue with 2.3, establishing the desired positive cognition.

B. Self-esteem and Guilt

If you suspect that the image triggers a negative view of themselves, repeat your question:

"What makes this picture evoke so much disturbance now?"

If the answer does not directly relate to a dysfunctional self-evaluation, but relates to a specific behavior: 'That I stood by and did nothing'), then the following questions help:

- *"What does that say about you as a person (that you didn't do anything, or is it about who or what you are?")*
- *"What is such a person often called? /What would you call such a person?"*

If it remains unclear to them, the following question might help:

- "When you look at this picture, what negative statement about yourself comes to mind the most right now?"

Check:

If there are several possible NC's and they have difficulty choosing:

"Look at that picture and say in your mind 'I am... (NC1). *'Keep looking at the picture and now say 'I am....* (NC2*).' Which of those two statements feels the strongest to you about the picture? "*

NC:

2.3 Desired (positive) cognition (PC)

If chosen for standard NC:

"The goal of this therapy is that you can look at the picture without feeling disturbance and that you can handle it."

If they have chosen a different NC, then formulate a PC that is directly opposite, for example:

When you bring up that image, what would you like to believe about yourself, instead of .... [NC]. ....*?"*

- ❑ Safety: (concerns the situation): "I am (now) safe"
- ❑ Self-esteem: "I am okay, worthwhile, competent, strong" or "I am good".
- ❑ Responsibility/Guilt: "I did what I could" or "I'm innocent" or "I've learned from it."

Helpful questions:

"What do you call someone like that who...?"

"Do you know someone who isn't... [NC]*?"*

"What do you call people who... ?"

PC: [ ]

2.4 Validity of Cognition (Voc 1-7)

When you bring up that picture (or experience) , how true do those words...[PC]..... *feel to you now on a scale, where 1 means 'false' and 7 means 'completely true'?"*

VoC [ ]

2.5 Emotion

"When you bring that picture to mind with all the other aspects that go with it: the sounds, smells, tastes and physical sensations - and the words 'I can't handle it'/'I can't stand it' (or other NC), what emotion do you feel now, right now?" (If necessary, offer suggestions: 'for example, scared, angry, sad'.)

2.6 Disturbance (SUD 0-10)

"Bring up the image and say to yourself...[NC]..., how disturbing does it feel right now, at this moment, estimated on a scale from 0, no tension at all, to 10, maximum tension?"

If the SUD is 5 or lower, ask them to activate the triggers in that memory network (visual, auditory, kinesthetic, olfactory and gustatory).

"Consider all sensory aspects that belong to the picture, including sounds, smells, tastes and physical sensations."

2.7 Location of the disturbance

"Where in your body do you feel that disturbance most strongly?"

3. Desensitization

Because the method is based on working memory theory, this means that the person is asked to try their best to hold the memory as long as possible, while the therapist tries to interrupt this attention focused on memory ('disruption').

a. *"So now please focus on the most disturbing aspect of this memory keeping all the details in mind. Ok, do you have it? And I'm going to distract you as much as I can. Think of it as a kind of competition: you will think about it as much as possible, and I will distract you as much as possible. Let’s see who wins. I think it’ll be me! ☺....*

   Let's get started quickly. Bring the image to mind, say to yourself: 'I can't handle the picture'/'I can't stand the image' [or other NC]. Be aware of the distress in your [location of the distress]. "

Give them a moment to focus.

"Start tapping first… Ok. And now follow my hand and open yourself up to whatever spontaneously comes to mind next."

Conduct a set with super-fast eye movements at 10 cm away from their face (just in front of the nose) for about half a minute.

With EMDR 2.0, many different tasks are used to overload the working memory, almost always in combination with each other (headphones plus buzzers plus games etc.). Therefore, always start immediately with complicated taps and super-fast eye movements. Then add more working memory taxation in the next set with one of the following working memory-taxing tasks, which you can alternate:

1. Diagonal eye movements, top/bottom, sideways, parabolas, circles, and 'signatures' [visual]
2. Representations (counting/spelling: alphabet forwards and backwards, words and sentences) [visual].
3.'Tik-tok' (or: 'Ticky -Tocky'). Potentially complemented by talking through it [auditory],
4. Fear inducing and disruptive/arousal-enhancing techniques (eg. 'blowing', whoosh!') [auditory]
5. V-step [kinesthetic] + combinations

b. *"What comes up?" /"What's going through your mind?" /"What do you notice?"*
(don't start a dialogue!) ....

Remember: that after going back to target, you can tax in modality-specific ways. Apply this consistently to match the modality someone mentions (for example: "I still hear my father crying”: auditory taxation).

Taxation Modalities

• Auditory (for example: singing a song, humming),

• Olfactory (for example: smelling types of tea, perfume, ammonia),

• Gustatory (for example: eating candy).

• Kinesthetic (for example: tapping using more difficult or faster patterns, or the V-Step)
"Focus on that"/"Concentrate on that."

Conduct Set with Distracting Stimulus

Continue until no new changes occur ('end of an association chain'). In principle, return *to target* every 5 – 10 minutes.

Return to Target

a. Test SUD.

Bring back the image we started with, as it is now stored in your head. How disturbing does it feel now on a scale or 0 to 10, where 0 means no disturbance at all or completely neutral, and 10 means the most disturbance possible," (WITHOUT USING THE NC!).

If SUD > 0:

b. *"Which aspect of that image is causing the most disturbance at this moment?"*

(Possibly *mentioning the SUD rating. For example: "What in particular makes that a (4)?")*

What do you see?

c. "Focus on that aspect................. Ok, do you have it?"

Conduct another Set with the distracting stimulus (until there is no more change, and within 5-10 minutes, return to a: '*Back to Target'*.)

Repeat the '*Back to* Target' procedure until SUD = 0 is reached for the target:

"*Are you absolutely sure that there isn't even a very small amount of disturbance left when you look at the image?...... allow yourself to feel it.........* "

If necessary (i.e., SUD > 0) continue with desensitization.

Once the SUD = 0 for the picture, check the whole 'video' or 'photo album to determine whether there really are no disturbing pictures left in the memory:

"*You indicate that this image no longer causes disturbance. Now please close your eyes and look at the entire memory again, as it is stored in your head at this moment. Are there any pictures in this memory that still cause you distress?"*

If the session cannot be completed with SUD = 0, then conclude in a positive way (step 6).

4. Installation of the PC

a. Test VoC (with PC + image)

Bring back the original image, the image we started with, as it is now stored in your head, and say to yourself 'I can handle the picture now/I can now stand the picture '[or other PC]. *On a scale of 1 (completely false) to 7 (completely true), how true does that statement feel?*

b. Instructions (if VOC reaches 7):

"Look at the image and say in your mind 'I can handle the picture now/'I can now stand the image'[or other PC] *Right! Do you have that?"*

c. Conduct a Set with distracting stimulus.

Always do a set of super- fast eye movements. Don't ask about associations! Continue directly with a. Keep going (a, b & c) until a VOC of 7 is reached.

5. Body scan (Only if you still have enough time left in the session)

a. *"Close your eyes, bring the image (image or picture or memory?) to mind, say to yourself 'I can handle it now/I can stand looking at the image(picture) now/I can deal with it*' [or another PC] *and pay attention to your entire body, from head to toe and notice if there is any physical tension anywhere connected to this image."*

b. In the case of remaining disturbance: Conduct a set with distracting stimulus (super-fast eye movements) and ask, *"What comes up now"?* Then continue following their associations (with continued desensitization). If no new associations arise, continue with a) and b) until the disturbance is gone and/or there are no new associations.

If disturbing images emerge regarding associated feared events, and certainly in the case of avoidance behavior including avoidance behavior due to anticipatory fear, then desensitize this with the "*Flashforward"* technique and, if useful, the "*Mental Video Check" and "Future Template".*

6. **Positive Closing** (at the end of each session; independent of target)

"What is the most positive or valuable thing you have learned (or experienced) about yourself in the past hour regarding...

(this theme; this memory; the last session)?"

Use one of these options if useful*:*

a. *"What does that say about you (as a person)?"* or *"What do you call someone like that?"*
b. " *Posture yourself like someone who is.....”*

"Just notice." *Focus for about 10 seconds on the positive self-talk, the associated positive body posture.*

b After about 10 seconds of concentrating, *"Is there anything else positive that comes up?"*

e. Continue processing to check if any more positive associations come up. Then, focus again on the positive association, for about 10 seconds, until no further positive changes occur.

Give directions about the upcoming (three) days, appointments: keeping a diary, your accessibility, etc.

7. Follow Up Session

a. If SUD > 0 at the end of the previous session: check it again at the start of this session. Choose from the following options:

- If SUD > 0 at the start of the session: begin the desensitization with *Back to Target* (without mentioning the NC).
- If SUD for the target = 0: check the entire 'video’ or 'photo album' to ascertain whether there really are no more pictures in the memory that evoke disturbance.
- If the SUD scores from all the targets associated with the memory is 0, and the VOC for the PC for the first chosen target is 7, you can:

- complete treatment if the intended result of therapy has been achieved, or
- continue with any subsequent identified memory (or memories) or

adjust the treatment plan based on the result achieved and continue the treatment.
