# Supplementary material for: The effectiveness, efficiency, and acceptability of EMDR vs. EMDR 2.0 vs. the Flash technique in the treatment of patients with PTSD: study protocol for the ENHANCE randomized controlled trial
Source: Front Psychiatry. 2023 Nov 9;14:1278052. doi: 10.3389/fpsyt.2023.1278052 (PMC10665892; doi:10.3389/fpsyt.2023.1278052)
Supplement: Supplementary file 2 [file Data_Sheet_2.pdf]

Shapiro's EMDR Standard protocol

Fase II: Preparation:

Give an explanation about EMDR and give specific instructions:

''When a trauma occurs it seems to get locked in the brain with the original picture, sounds, thoughts, and feelings. Since the experience is locked there, it continues to be triggered whenever a reminder comes up. It can be the basis for a lot of discomfort and sometimes a lot of negative emotions, such as fear and helplessness, that we can't seem to control. The eye movements we use in EMDR seem to unlock the system and allow the brain to process the experience. That may be what is happening in REM or dream sleep. – the eye movements may help to process the unconscious material. It is important to remember that it is your own brain that will be doing the healing and that you are the one in control.''

''Disturbing events can be stored in the brain in an isolated memory network. This prevents learning from taking place. The old material is repeatedly triggered. In another part of your brain, in a separate network, most of the information you need to resolve it is stored. It's just not possible to connect these two networks. Once we start the processing with EMDR, the two networks can connect. New information may come to you that helps process the experience.''

''What we will do several times during this procedure is to check what comes up for you or goes through you. Please tell me as clearly as possible what is happening. Sometimes things change, and sometimes they don't. I will occasionally ask you how much tension you feel on a scale of 0 to 10, and sometimes it changes, and sometimes it doesn't. I may ask if something else comes up for you, and sometimes it does, and sometimes it doesn't; there's no specific way it should go. Please just tell me as clearly and accurately as possible what is happening to you without wondering what should happen. Just let it happen naturally.''

Establish a stop signal:

''If there's a moment when you feel that we should stop, please raise your hand.''

Introduce the eye movements to the client:

Offer the distraction at a pace that the client can just keep up with.

Establish a metaphor for the associations:
‘’Perhaps you can imagine it's like sitting in a train and just observing what's happening or passing by around you, outside the train. As you look at the scenery, you notice your thoughts, feelings, and bodily sensations as they go by. Alternatively, you may prefer to imagine it's as if it's only projected on a movie screen. You just have to watch it. It's old material, a memory. It's not happening now.’’

Emotion regulation (only during the first session):
Explore and install the safe place. Install resources as needed. If the client is unable to access a sense of safety due to the traumas, look for a place where the client can feel calm or at ease or a place that invokes another positive feeling.

‘’Can you imagine a safe place, one that's easily accessible and provides a personal sense of peace and security? (Give the patient time to concentrate) Now, focus on this image. Feel the emotions and be aware of where you can feel the pleasant sensations in your body.’’

If necessary, use soothing sounds to enhance the imagination and the pleasant feeling. Then, encourage the patient to experience the pleasant feeling of the safe place:

"Take the image of the place that feels peaceful and safe/calm in your mind. Focus on the place in your body where you perceive the pleasant feeling and allow yourself to enjoy it. Now, concentrate on those feelings as you follow my fingers with your eyes."

Short set of slow eye movements (4 to 8 movements). Inquire about the feeling it evokes.
"How do you feel now?"

If this pleasant feeling intensifies: Continue with sets of eye movements as long as the pleasant feeling continues to increase. Establish a keyword that best describes the image of the safe place (e.g., beach, mountain, relaxation) and install it with slow eye movements. Encourage the patient to evoke the pleasant feeling:

"Which word best describes the image of the safe place? Repeat this word in your mind as you look at the image of the safe place and notice the pleasant feelings."

Set of slow eye movements. Repeat this procedure four to six times as long as the pleasant feeling continues to increase. Then, let the patient practice evoking the pleasant feeling on their own (without eye movements):

"Keep repeating (the keyword) and visualize the safe/calm place in your mind, experiencing the pleasant feelings."

When the client has successfully completed the exercise, instruct them on how to use it to relax when experiencing stress:

"Now, think of a situation, not too intense, that you found unpleasant. Bring up the uncomfortable feelings associated with this memory. Once you have those feelings, bring to mind the safe/calm place again, repeat (the keyword), and experience the pleasant feelings until the negative feelings disappear."

Now, let the client perform the exercise on their own.

"Now, bring the same unpleasant situation back to mind and re-experience the negative feelings. Perform the exercise as we practiced it earlier until the negative feelings disappear.’’

When the client successfully manages to perform the safe/calm place exercise, you can proceed to phase III.

Phase III: Assessment:

Ask for the memory to be processed:

"We agreed to work on the memory of .... today. Can you bring the memory to mind?"

Target selection:

''What picture represents the worst part of the memory as you think about it now?''

Negative cognition:

"Bring the image to mind. What negative thought do you have about yourself when you look at this image?"

Assistance: If the client provides a situation-specific statement, such as 'I couldn't do anything,' ask:

"What does that say about yourself, I am ..."

Desired (positive) cognition:

''When you bring up that picture, what would you prefer to believe about yourself?''

Validity of Cognition: (VoC 1-7):

''When you think of the memory, how true do the words (PC)….feel to you now on a scale from 1-7, where 1 feels completely false and 7 feels completely true?''

Emotion:

"When you bring the image to mind and simultaneously say (NC) to yourself, what kind of emotion do you feel?"

Disturbance (SUD 0-10):

''On a scale from 0 to 10, where 0 is no disturbance or neutral and 10 is the highest disturbance you can imagine, how disturbing does it feel to you now?''

Location of feeling:

''Where do you feel it, the disturbance, in your body?''

Phase IV: Desensitization:

Remind the client (optional) that it's their own brain that is doing the processing. Let whatever comes up come up. If they would like to stop, raise a hand.

‘’Bring up that image, together with those negative words (repeat NC), notice where you are feeling it in your body, and follow my fingers…’’

Begin the eye movements. As a rule of thumb, start with a minimum of 24 sets of eye movements at the beginning. Afterward: Observe the client and extend the set if the client needs more time to process.

If strong emotions arise: Provide encouraging statements during the set to help continue with the processing, such as 'let it come, it's a natural part of it, they are memories, you are safe now.'

If you notice changes in facial expression, emotion, etc., ask the client to take a deep breath and stop the eye movements. Then, ask: "What do you notice now?" or "What comes up?"

As long as there are changes (movement), say: "Notice that" or "continue with that."

If negative self-statements come up as feedback, ask the client: "What do you notice in the body? Observe that."

Continue with the eye movements.

If the client's feedback indicates that the train is still moving (new information, insights, images, emotions, etc.), continue with the eye movements.

If the client provides two consecutive sets of positive or two neutral feedback responses, then go Back to Target: "Go back to the (complete) memory. What do you notice now?"

Wait for the client's feedback. Then: "Notice that" or "continue with that."

Begin the eye movements. Have the client follow their associations.

After sufficient processing (going through multiple association channels and no new material emerging): Only when there's a suspicion that SUD = 0: "Bring the original memory to mind. On a scale of 0 to 10, where 0 means no tension/neutral, and 10 means as much tension as possible, how much tension do you feel?"

If SUD = 0, proceed with installing the PC.

If SUD > 0, continue with processing. If the SUD remains at 1 or 2, ask: "What is needed to bring it to 0? Possibly: where do you feel that (the tension at 1 or 2) in your body?" Continue until SUD = 0.

In case of blocking or looping:
If there are signs of blocking or looping, go Back to Target and/or consider changing the speed or direction of the eye movements, or introduce a different distracting task instead of eye movements.

If the above does not resolve blocking or looping, then consider using a cognitive interweave.

If that has no effect, consider using a floatback.

Floatback:
"Hold the image and (NC) in your mind, notice the physical sensations in your body, and let your thoughts drift back to an earlier moment in your life. What is the first moment that comes to mind where you also felt this?"

If an earlier memory (feeder memory) comes up, consider it as an association within the processing of the original target. Once the association channel has been traversed, return to the original target.

Phase V: Installation of PC:

Check the PC: "When you bring the memory back to mind, do the words (PC) still fit, or do you find another positive belief about yourself more appropriate?"

Validitity of Cognition PC (VoC 1-7):

"Bring the memory to mind and say to yourself (PC). On a scale of 1 (completely untrue) to 7 (completely true), how true does it feel now?"

Installation of the new PC with the original event:

"Bring the memory to mind and say to yourself: 'PC'."

Start eye movements. Repeat the question about VoC (see above).
Continue this until VoC = 7.

If VoC no longer increases, ask: "What is needed to reach a 7?"

If the client now identifies a dysfunctional belief such as, "I don't deserve to be healthy," which doesn't disappear with multiple sets of eye movements, investigate which memory is driving this belief and process it as at the beginning of Phase 4. If necessary, use a floatback to identify the memory.

Sometimes, adding a "softener" to the PC can help achieve a VoC of 7, for example, "I can come to believe that I...". This way, the original PC doesn't have to be completely lost.

When a non-problematic statement is made, such as: "I need to give it time to be sure this belief can be true," proceed with the body scan.

Ensure that the client links the VoC to the memory, not to the future.

Floatback:

"Hold the image and the (dysfunctional belief) in your mind, notice the sensations in your body, and let your thoughts drift back to an earlier moment in your life. What is the first moment that comes to mind where you also felt this?"

If the VoC = 7, proceed with the Body Scan.

Phase VI: Body scan:
"Close your eyes. Think of the memory and the positive words (PC) and mentally scan your entire body from head to toe. Check if there are still any physical sensations somewhere."

Set of eye movements (always). Repeat the question in the above box. If positive sensations are reported during the Body Scan and they become stronger, do a set and repeat the Body Scan until these sensations no longer increase.

If the feedback from the Body Scan yields negative physical sensations, do a set of eye movements and repeat the Body Scan as described above. Repeat until the Body Scan no longer yields results.

Ensure during the Body Scan that the physical sensations the client reports are not related to an existing physical condition or other memories that still need to be worked on.

Phase VII: Closure:
Two types of closure:

1. A completed session (SUD = 0), VoC = 7, Body Scan finished:

"It is possible that what we've worked on today may continue to work after the session. You may have new insights, thoughts, memories, or dreams."

2. An unfinished session (SUD > 0, in Phase 4, 5, or 6):

"You've worked very hard today. It is possible that what we've worked on today may

continue to work after the session. What have you learned from this session that can be helpful for you in the coming week?"
