# Supplementary material for: The effectiveness, efficiency, and acceptability of EMDR vs. EMDR 2.0 vs. the Flash technique in the treatment of patients with PTSD: study protocol for the ENHANCE randomized controlled trial
Source: Front Psychiatry. 2023 Nov 9;14:1278052. doi: 10.3389/fpsyt.2023.1278052 (PMC10665892; doi:10.3389/fpsyt.2023.1278052)
Supplement: Supplementary file 3 [file Data_Sheet_3.pdf]

FLASH PROTOCOL

1. Introduction

When the Flash technique is used, the symptoms are known, and the memory to be worked on has been identified. For this procedure, select a memory that triggers the symptoms; that is, a memory that is unrelated to and does not connect with other negative memories that have occurred before.

"Today, we will work on your unpleasant memory. The procedure we are about to perform requires you to recall a positive memory and focus on it. I will ask you to select a positive memory in a moment. As for the negative memory, you don't need to think about it often. I will only ask you to think about it briefly at the beginning, so I can determine how disturbing the memory is for you. Furthermore, I will ask you that question a few times during the session. This recall or retrieval of the memory should be very brief. Only if I occasionally check it, I know whether the memory still evokes tension or disturbance. So, during the session, you will mainly focus on pleasant, positive things. Shall we begin?"

2. Check SUD Score

"I would like to ask you to briefly recall the negative memory you have selected. When you think about this memory, how disturbing does it feel or how much tension do you feel on a scale from 0 to 10, where 0 is no disturbance or neutral and 10 is the highest disturbance you can imagine, how disturbing does it feel to you now?''

SUD SCORE:

3. Identifying positive engaging focus

"Okay... Now, you can set aside that memory for a moment. We are about to perform a procedure in which I will ask you to recall a positive memory. Ideally, it should be a memory of an activity, such as sports, walking, skiing, or dancing. However, it can also be a positive memory of a loved grandmother, a pet, or a family member, or perhaps a beautiful journey or another extremely positive memory. It can also be a memory of a situation that still makes you laugh. Can you select a memory? Okay, tell me..."

Allow the patient to share and check if the memory elicits enough positive emotions (non-verbal signals or verbal confirmation).

"I can see that this memory is making you smile. Very good."

"I'm going to ask you to recall the positive memory as vividly as you just did, but this time, I'm going to ask you to 'flash' as well, which means blinking your eyes five times in a row. Can you rapidly blink your eyes five times? It's important that you do this without thinking about the negative memory. This works best when you are fully immersed in the experience of the positive activity or memory and not thinking about the negative memory. Alright, let's get started!"

4. Focussing

"First, I'd like you to recall the pleasant memory (mention a few keywords). If you wish, you can close your eyes. Bring this positive memory to mind as vividly as possible."

You can enhance the person's experience by asking a question, for example:

"What are you doing/where are you/who are you with? And can you vividly imagine yourself (doing...)? What else do you see/hear/feel/smell/taste?"

Stimulate all sensory perception and prolong the feeling as much as possible.

"Yes, feel how wonderful this is."

Do this for approximately 20-30 seconds and check with the patient if he/she has the memory vividly in mind.

"Do you have this pleasant memory clearly in your mind? How pleasant would you rate this memory now from 0 to 10?"

SUD Score for Positive Memory:

5. Flash

"Recall this positive memory as vividly as possible. Are you fully immersed in it?"

If the patient is not fully immersed, ensure that they are and provide cues to maximize the experience (people, places, sounds, sensations, ask questions).

Ready? "Flash!"... (if someone doesn't respond: 'Rapidly blink five times in a row'). 'Stay entirely focused on your positive memory; 'You're completely in it.'

Flash!, (wait for the patient to do it)

Flash!, (wait for the patient to do it)

Flash!, (wait for the patient to do it)

Flash!, (wait for the patient to do it)

During the breaks, make sure the patient stays fully engaged in the experience by encouraging them:

"Yes, that's good." "Are you fully immersed again?" "Think of the most enjoyable moment." "Visualize it again." "Feel it." "What's the most delightful part?"

6. 'Back to Target'

The intention is to briefly return to check the distress level of the negative memory after 5 'flashes'.

'Alright, excellent! Now, think about that negative memory again (give the patient a moment). What has changed? How disturbing does it feel or how much tension do you feel on a scale from 0 to 10, where 0 is no disturbance or neutral and 10 is the highest disturbance you can imagine, how disturbing does it feel to you now?'

The intention is to spend little time exploring the negative memory. The return to the negative memory is only meant to determine SUD (and not to have the memory described again). If the score hasn't decreased, try to clarify what might have gone wrong.

- Did the patient blink firmly enough?
- Or maybe they thought about the negative memory in between?

If there is residue (SUD > 0), repeat the steps from '5. Flash Positive Memory.' Continue with the Flash! (return to the positive memory) until the patient no longer indicates any distress.

7. Final Check

"Okay, just to be sure, recall the negative memory again. When you think about this memory, how disturbing does it feel or how much tension do you feel on a scale from 0 to 10, where 0 is no disturbance or neutral and 10 is the highest disturbance you can imagine, how disturbing does it feel to you now?’

If there is residue (SUD > 0), repeat the steps from '5. Flash Positive Memory.' Continue with the Flash! (return to the positive memory) until the patient no longer indicates any distress. Then, recheck with the final check question.
